# Hideyo Noguchi African Prize Promoting Medical Research and Medical Service to Fight Infectious Diseases in the Africa

**DOI:** 10.29245/2578-3009/2021/S2.1106

**Published:** 2021-04-15

**Authors:** Hideyo Noguchi

After extensive travel throughout Central and South America researching on vaccines for such diseases as yellow fever, Oroya fever and poliomyelitis which threatened the lives of millions of people in those days, he eventually ventured into Africa to confirm his findings. He tried to demonstrate the hypothesis that yellow fever was caused by spirochete bacteria but in vain, because at that time the electron microscope to observe viruses had not been invented yet.

While working in Accra, Ghana, he was struck down by the yellow fever virus, his last words being “I don’t understand.” The grave of Dr. Hideyo Noguchi in Woodlawn Cemetery in New York, the United States, is inscribed with the following epitaph: “Through devotion to science, he lived and died for humanity.” In 2004, Dr. Noguchi became the first Japanese scientist to have his portrait printed on a Japanese banknote (1000-yen note).

**Figure:**
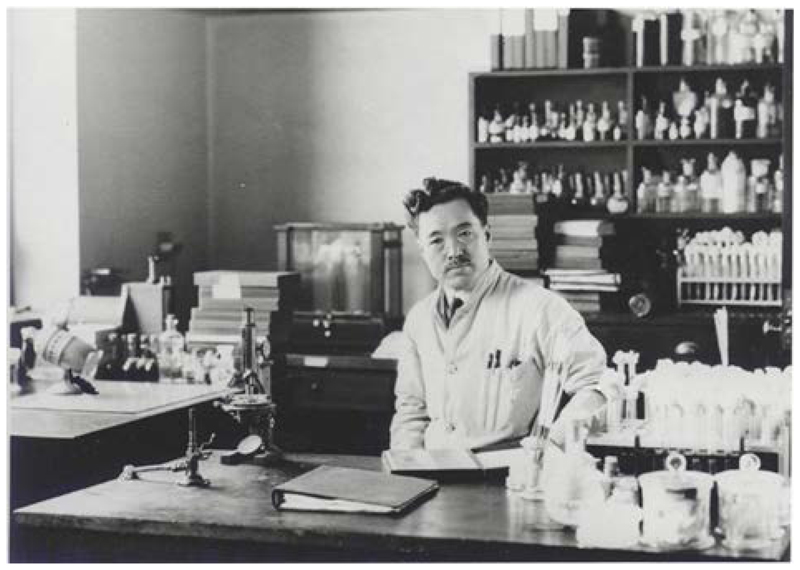
Dr. Hideyo Noguchi in his lab at the Rockefeller Institute for Medical Research (Photo: Hideyo Noguchi Memorial Foundation)

**Figure:**
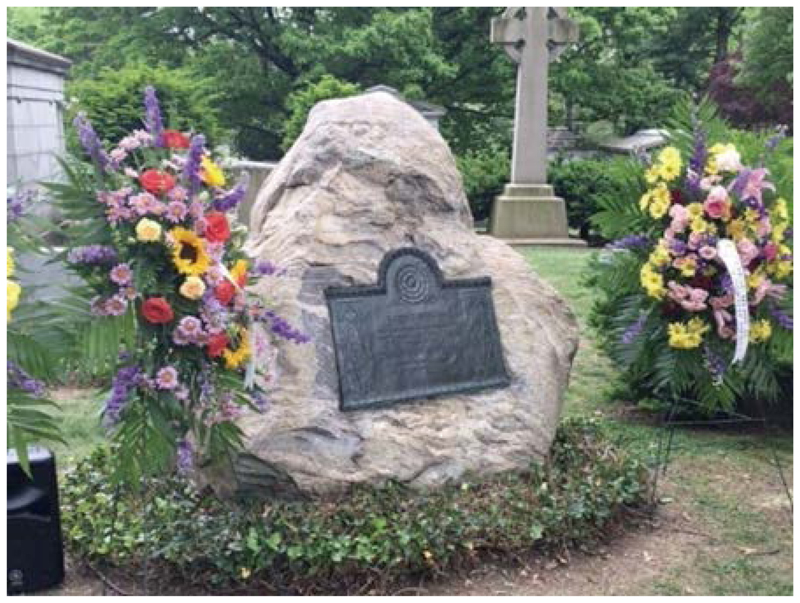
The grave of Dr. Hideyo Noguchi in Woodlawn Cemetery (Photo: New York Hideyo Noguchi Memorial Society, Inc. (HNMS))

## What is HIDEYO NOGUCHI Africa Prize?

The spread of infectious diseases presents a common threat to all. Mindful that Africa faces this scourge most acutely, the government of Japan established the Hideyo Noguchi Africa Prize (HNAP) in July 2006 in memory of Dr Hideyo Noguchi (1876-1928) whose belief in medical advancement and self-sacrificing activities in Africa remains a beacon of inspiration to all.

Guided by these ideals ad mindful of the human suffering persisting in Africa, the region facing the most serous health challenge on the globe, the Prize aims to honour individuals with outstanding achievements in the fields of medical research and medical services to combat infectious and other diseases in Africa, thus contributing to the health and welfare of the African people and of all humankind. The prize for medical services in coordinated in a collaboration of the WHO Regional Office for Africa and the Ministry of Health, Labour and Welfare of Japan.

Winners of the first three sets of the Hideyo Noguchi Africa Prize for Medical research and medical services are as follow:

**Table:** 

Prize Year	Medical Research	Medical services
2008	Brian GREENWOOD ***(United Kingdom)*** Professor of Clinical Tropical Medicine London School of Hygiene and Tropical Medicine, UK	Miriam K. WERE ***(Kenya)*** Dr of Public Health, Health Planning and Management Co-Founder, and Health Specialist UZIMA Foundation, Kenya
2013	Dr Peter PIOT ***(Belgium)*** Director and Professor London School of Hygiene and Tropical Medicine, UK	Dr. Alex COUTINHO ***(Republic of Uganda)*** Executive Director Infectious Disease Institute Makerere University, Uganda
2019	Dr Jean-Jaques Myembe-Tamfum (***Democratic Republic of Congo***) Professor of Medical Microbiology/Virology, Faculty of Medicine, University of Kinshasha General Director of the National Institute of Biomedical Research (INRB).	Dr Francis Gervase Omaswa (***Republic of Uganda**)* Executive Director of African Centre for Global Health and Social Transformation (ACHEST)

### - Excerpt from the pamphlet of Hideyo Noguchi Africa Prize

From the array of laureates, one finds personalities that had accomplished themselves in the different areas of their training and expertise. The quality participation in future competitions will depend largely on the ability of current generation of scientists and activists to begin now in preparing themselves. It is in this wise that the HNAP is supporting the mentorship of young scientists to begin to cultivate the virtues of the past laureates in hard work, diligence and scientific writing. The financial support provided to this publication is thus a movement that will set the HNAP as a goal for the youth.

